# A Modified Warner and Fackler Technique With Posterior Plate for Kyphectomy in a Myelomeningocele Patient With Lumbar Kyphosis

**DOI:** 10.1155/cro/7972090

**Published:** 2026-04-16

**Authors:** J. Manuel Sarmiento, Saagar Dhanjani, Nitin Jagdhane, Ryan Goodwin, Anthony S. Rinella

**Affiliations:** ^1^ Pauline Braathen Neurological Center, Department of Neurosurgery, Cleveland Clinic Florida, Weston, Florida, USA, clevelandclinic.org; ^2^ San Diego Spine Foundation, Department of Research, San Diego, California, USA; ^3^ Cleveland Clinic Foundation, Department of Orthopedic Surgery/A4 1, Cleveland, Ohio, USA, clevelandclinic.org; ^4^ Duly Health and Care, Naperville, Illinois, USA

**Keywords:** gibbus deformity, kyphectomy, lumbar kyphosis, myelomeningocele, Warner and Fackler technique

## Abstract

Congenital kyphotic spinal deformities in children with myelomeningocele are usually progressive and can impair sitting posture. Kyphectomy is classically performed to restore spinal alignment, but the procedure is historically associated with high complication rates. There are several variations of kyphectomy surgery. We favor an all‐posterior, modified Warner and Fackler procedure with plate fixation. A 5‐year‐old, nonambulatory female with a history of myelomeningocele repaired at birth presented with a progressive, semirigid 132° lumbar kyphosis to clinic during a surgical mission trip to Colombia. The lumbar kyphosis was increasingly impairing her sitting posture and balance. We indicated this patient for kyphectomy surgery involving an all‐posterior, modified Warner and Fackler procedure with long S‐shape rods cantilevered against the anterior sacrum and posterior plate fixation for spinal fusion and deformity correction. A modified Warner and Fackler procedure with long S‐shape rods anchored in the first sacral foramen and cantilevered against the anterior sacrum with posterior plate fixation is our preferred technique for kyphectomy in myelomeningocele patients with lumbar kyphosis to restore spinal alignment and reduce lumbosacral instrumentation prominence.

## 1. Introduction

Myelomeningocele (MMC) is one of the most common congenital disabilities, affecting approximately 0.005% of live births [1, 2]. It occurs due to the incomplete closure of the caudal end of the neural tube during the fourth week of gestation, leading to the improper development of the posterior spinal elements [2]. The incomplete development of the posterior spinal elements, absence of a posterior tension band, and imbalance of paraspinal musculature contribute to progressive kyphosis in 12%–28% of children with MMC [3]. This progressive kyphosis often culminates in the development of a gibbus, a sharp angular deformity typically located between L2 and L5 [4, 5]. The presence of a gibbus is associated with several significant comorbidities, including skin breakdown over the apex of the kyphosis, impaired sitting balance that requires hand support for stability, and severe back pain that prevents the child from lying supine [1].

Since casting, bracing, and wheelchair inserts provide minimal control of gibbus deformities, corrective surgery is often necessary [1]. The goals of surgery are reducing the spinal deformity, restoring spinal alignment, promoting healing of skin ulcerations, improving access to the abdomen for gastrointestinal or urinary diversion, enhancing sitting balance without hand support, enabling the patient to lie supine, and improving cosmesis [5].

Traditionally, a rigid congenital kyphosis in MMC has been managed with kyphectomy followed by a posterior instrumented fusion. However, this procedure is associated with a high rate of complications, including pseudoarthrosis, loss of correction, infection, wound breakdown, and instrumentation prominence [6–8].

In light of these challenges, the authors report this case of a young patient with MMC who underwent a posterior kyphectomy combined with a modified Warner and Fackler technique, which includes lumbar pedicle screws, long S‐shaped rods secured through the first sacral foramina to rest against the anterior sacral cortex, and a posterior plate [9].

## 2. Case Report

**Figure 1:**
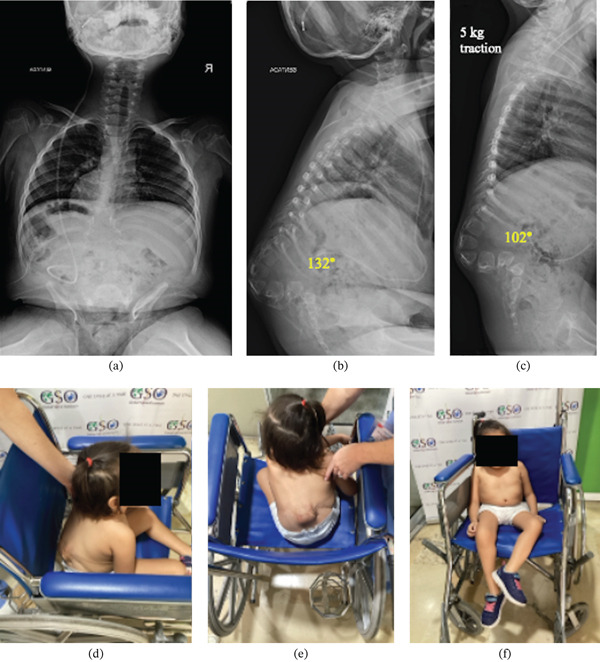
(a) Preoperative AP, (b) lateral sitting, and (c) lateral sitting with 5 kg of Gardner‐Wells gravity traction. Clinical photos of patient sitting in the (d) lateral view, (e) posterior view, and (f) front view.

A 5‐year‐old, nonambulatory female with a progressive, semirigid 132° congenital lumbar kyphosis presented to clinic during a surgical mission trip to Colombia. Her past medical history was significant for MMC that was repaired at birth and hydrocephalus for which a ventriculoperitoneal shunt (VPS) was placed. The lumbar kyphosis was increasingly impairing her sitting posture and balance. On physical examination she measured 2 ^′^9 ^″^ and weighed 33.62 lbs for a BMI of 21.36 kg/m^2^. Her shoulders are leveled, there is a lumbar paraspinal prominence, and she had a stooped forward, kyphotic sitting posture (Figure 1). Her bilateral lower extremities were not functional. She had a healed transverse lumbar incision with scar and granulation tissue.

Standing AP and lateral scoliosis radiographs revealed a 132° lumbar kyphotic gibbus deformity without concerning pelvic obliquity or scoliosis (Figure 1a–d). AP and lateral traction radiographs showed a semirigid lumbar kyphotic deformity, and the deformity reduced to 102° with 5 kg of Gardner‐Wells gravity traction (Figure 1). There was no available advanced imaging such as computed tomography or magnetic resonance imaging. We indicated this patient for a kyphectomy surgery involving an all‐posterior, modified Warner and Fackler procedure with long S‐shape rods cantilevered against the anterior sacrum and posterior plate fixation for spinal fusion and deformity correction.

**Figure 2:**
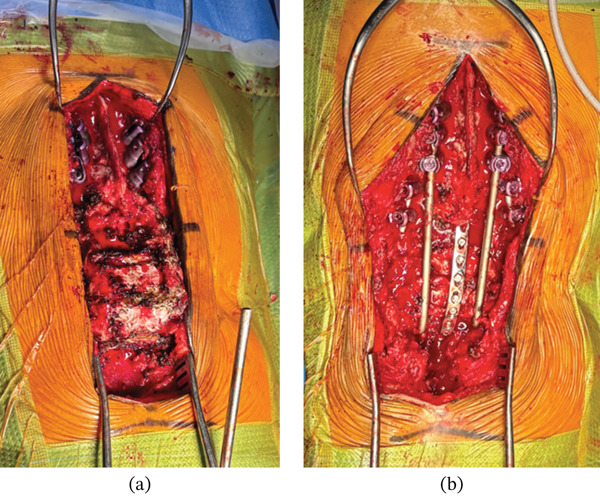
Intraoperative view of the lumbar kyphosis. We observed anomalous lumbar pedicle formation and absence of posterior osseous structures such as lamina, pars, facet joints, and spinous processes (a). Two titanium, 4.5‐mm diameter rods were contoured into an S shape at the distal ends and placed into both S1 neuroforamen. The rods were rotated 180°, cantilevered over the distal thoracolumbar vertebrae, and engaged into the proximal pedicle screws at T9–11. Two satellite rods were placed into the T12 and L1 tulips for fixation because their extreme lateral‐to‐medial trajectories had disrupted the cadence of the proximal tulip heads. After the lumbar kyphotic deformity was reduced, a midline compression plate (Medtronic, Minneapolis, Minnesota) was screwed into the posterior lumbar vertebrae and sacrum from L1 to S1 (b).

**Figure 3:**
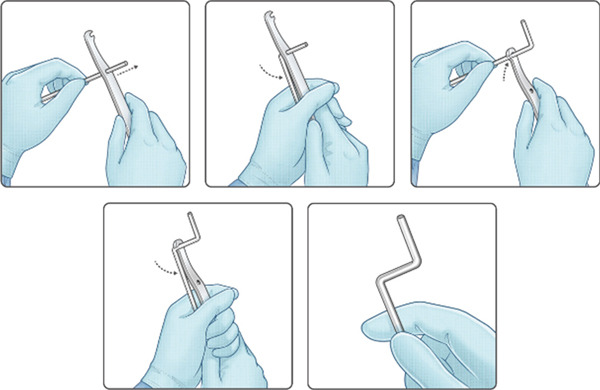
Step‐by‐step illustration of an intraoperative rod bending technique to create the S‐shaped rods.

**Figure 4:**
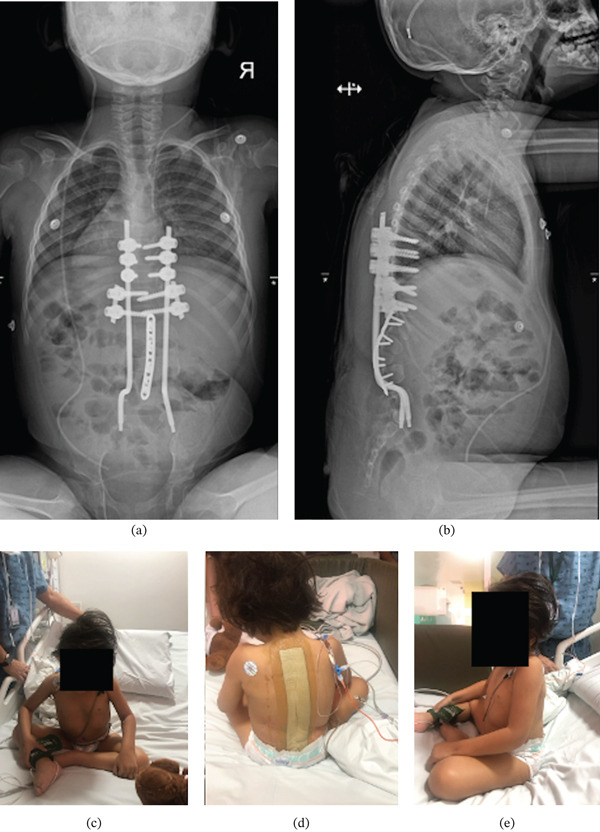
(a) Postoperative AP and (b) lateral sitting x‐rays. Postoperative clinical photos of patient sitting in the (c) front view, (d) posterior view, and (e) lateral view.

We performed a posterior, midline thoracolumbar approach to expose the midline thoracic spine to the sacrum. There was a rudimentary spinal cord under a sheet of scar tissue that was previously ligated at the T12–L1 level. During exposure of the lumbar kyphotic deformity, we observed anomalous lumbar pedicle formation and absence of posterior osseous structures such as lamina, pars, facet joints, and spinous processes (Figure 2a). The posterior wall of the lumbar vertebral bodies and their corresponding intervertebral disks were exposed. T9–11 pedicle screws measuring 4.0 × 25 mm were placed using free‐hand technique. Both T12 and L1 had anomalous and incompletely formed pedicles. Therefore, four pedicle screws measuring 4.0 × 30 mm were placed in a more lateral starting point and a medially angulated trajectory. Pedicle screw trajectories were checked with fluoroscopy for accuracy. Note that this medial trajectory leads to crossing screw tips on AP radiographs. A thorough discectomy was performed in the last four caudal levels from L1–L5. The endplates were denuded of their cartilage attachments, and the anterior annulus was packed with local autograft bone graft and recombinant human bone morphogenetic protein‐2 (rhBMP‐2; Infuse, Medtronic, Minneapolis, Minnesota) to enhance spinal fusion. Two titanium, 4.5‐mm diameter rods were contoured into an S shape at the distal ends and placed into both S1 neuroforamen (Figure 3). The rods were rotated 180°, cantilevered over the distal thoracolumbar vertebrae, and engaged into the proximal pedicle screws at T9–11. Two satellite rods were placed into the T12 and L1 tulips for fixation because their extreme lateral‐to‐medial trajectories had disrupted the cadence of the proximal tulip heads. After the lumbar kyphotic deformity was reduced, a midline compression plate (Medtronic, Minneapolis, Minnesota) was screwed into the posterior lumbar vertebrae and sacrum from L1 to S1 (Figure 2b). Estimated blood loss was approximately 200 cc, and operative time was 4 h and 40 min. There were no immediate postoperative complications. Postoperative sitting x‐rays showed good correction of lumbar kyphosis in the sagittal plane, and clinical photos demonstrated improved sitting posture (Figure 4). Sagittal and coronal spinal alignment were maintained at 1 year postoperative follow‐up without evidence of rod fracture, and the incision had healed properly without signs of infection.

## 3. Discussion

Rigid congenital kyphosis in MMC necessitates kyphectomy since nonsurgical treatments are ineffective in controlling the gibbus. Initially described by Sharrard et al. in 1968, the kyphectomy procedure has undergone several iterations, first developed from in situ fusion with spica casting to the modern version with instrumented fusion [10]. In spite of the advances in spinal instrumentation over the last several decades, kyphectomy remains associated with high morbidity and complications, including bleeding, infections, and even risk of sudden death due to intrathecal pressure disorders [6, 11–13]. Surgical indications for gibbus deformities in children include lumbar kyphosis exceeding 50°–70°, rapid curve progression, or development of new lumbosacral neurological deficit [14, 15]. Given the high risks of surgery in these complex patients, several important clinical factors need to be considered before surgical intervention: Skin condition, nutritional status, respiratory function at baseline, VPS patency, and urinary culture screening are some of the most important. Skin integrity, often compromised by pressure sores, requires careful preoperative assessment and plastic surgery interventions if necessary [6, 16]. Nutritional optimization, including parenteral feeding, supports wound and bone healing, whereas a patent VPS is essential to prevent cerebrospinal fluid pressure spikes during surgery [1]. Comprehensive counseling with the parents is crucial and helps set realistic expectations for this life‐improving but complex procedure.

## 4. Overview of Surgical Techniques

Several surgical techniques were proposed in the 1970s and 1980s that described correction and stabilization of lumbar kyphosis. Spinal osteotomy with vertebral body excision and anterior strut grafting were the most popular techniques, with Harrington rods and wire loops serving as the most common fixation methods. In 1987, Heydemann and Gillespie reported promising results with segmental instrumentation using Luque rods in 12 children with MMC kyphosis. In 1993, Warner and Fackler compared clinical outcomes between MMC patients with lumbar kyphosis using two different instrumentation techniques. Twenty‐one patients had Harrington compression instrumentation, and 12 patients had Luque rod instrumentation, with fixation through the first sacral foramina with a modification of the Dunn–McCarthy technique [9, 17]. This simple modification made a lateral bend in the rod unnecessary since the first sacral foramina are aligned with the lumbar foramina, and the rods are bent so that their distal tips hug the anterior surface of the sacrum. Sagittal contouring of the rods above the sacral bend is unnecessary because the rods lever the sacrum into extension and produce a lumbosacral lordotic angle that prevents a pressure point. Most importantly, the Warner and Fackler technique led to a more significant decrease in average lumbar kyphosis, a higher rate of postoperative fusion without any kyphotic deformity recurrence (100% vs. 62%, respectively), and a lower rate of postoperative wound infection (8.3% vs. 23.8%, respectively).

Other instrumentation methods have been used to provide lumbar kyphosis correction and stabilization. The Dunn–McCarthy S Rod Fixation Technique used contoured S‐shaped rods for pelvic obliquity correction by securing the rods to the sacral ala [18]. It fixes firmly against the sacral ala by distracting against a hook or pedicle screw at L5. However, the S‐shaped rods pose a risk of dislocation from the sacral ala secondary to pelvic rotation in patients with spasticity.

Another technique is the Ordent modification of the Dunn–McCarthy technique that placed the distal, S‐shaped rods through the first sacral foramina for lumbar kyphosis correction in the first stage. The second stage involved additional anterior tibial strut grafts for additional support. Our version of the kyphectomy procedure emphasizes securing fixation from an all‐posterior approach using a midline compression plate to avoid the attendant risks associated with an additional anterior procedure. We also favor additional satellite rod placement to address anomalous pedicle anatomy to avoid creating bends in the main correcting rods. These modifications provide satisfactory correction of lumbar kyphosis and render a previously technically challenging surgery into a much simpler one that can be reliably performed on surgical mission trips.

## 5. Surgical Outcomes

Kyphectomy for patients with MMC is aimed at correcting spinal deformities, providing children with a stable, balanced spine, which leads to improvements in activities of daily living (ADLs), social status, and self‐image [19]. However, it is important to stress that this procedure carries a high complication rate, exceeding 50% in some series [11]. Common complications include instrumentation prominence, pseudarthrosis, infection, wound breakdown, and loss of correction [12, 20, 21]. Mortality is rare but can occur, mainly if a VPS is not patent or during anterior dissection if major organs are injured [1]. The rate of nonunion and instrumentation‐related complications deserves a special attention because they are amongst the highest in pediatric spine surgery. In the largest series of 77 patients, the instrumentation‐related complications were 29.8%, and there was a 22% rate of screw loosening and pseudarthroses [12]. As instrumentation loosens, secondary loss of deformity correction can happen in up to 30%–76% of patients [12]. Infection rates range from 20% to 43% [11, 12]. In 2004, Niall et al. reported a 79% (19 of 24 patients) risk of wound complications [22]. Eleven of these patients had chronic issues with instrumentation prominence and exposure. Six patients had delayed wound breakdown due to instrumentation prominence and ulceration. This may require the removal of instrumentation that can also lead to secondary loss of deformity correction. The measures we took to prevent infection were utilizing vancomycin powder after saline irrigation of the surgical cavity, and performing a meticulous closure of the separate muscle, fascial, and subcutaneous layers to prevent seroma accumulating within dead space areas. Another reason we prefer the Warner and Fackler technique is because it inherently keeps sacral spinal instrumentation as low‐profile as possible to prevent risks of prominence and ulceration. For instance, incorporating posterior plate augmentation provides additional fixation to the spinal construct without having to add another bulky dual long‐rod construct and the associated lateral connectors involved with this strategy. Our technique also obviates the need for placement of pelvic instrumentation and exposure of the iliac crests, which can be challenging to successfully close in these patients and risks prominence of iliac instrumentation through the skin. Despite these challenges, kyphectomy remains an effective solution for correcting gibbus deformities in MMC patients, offering pediatric patients a stable, balanced spine [1].

## 6. Conclusion

A modified Warner and Fackler procedure with long S‐shape rods anchored in the first sacral foramen and cantilevered against the anterior sacrum with posterior plate fixation is our preferred technique for kyphectomy in MMC patients with lumbar kyphosis to restore spinal alignment and reduce lumbosacral instrumentation prominence.

## Funding

No funding was received for this manuscript.

## Disclosure

This manuscript is original and has not been submitted elsewhere in part or in whole.

## Consent

Our patient′s parents were informed that data concerning the case would be submitted for publication and were in agreement for publication.

## Conflicts of Interest

The authors declare no conflicts of interest.

## Data Availability

Data sharing is not applicable to this article as no datasets were generated or analyzed during the current study.
